# Ischemic stroke as a complication of cryptococcal meningitis and immune reconstitution inflammatory syndrome: a case report

**DOI:** 10.1186/s12879-018-3386-0

**Published:** 2018-10-16

**Authors:** Jayne P. Ellis, Newton Kalata, Elizabeth C. Joekes, Samuel Kampondeni, Laura A. Benjamin, Thomas S. Harrison, David G. Lalloo, Robert S. Heyderman

**Affiliations:** 1 Malawi-Liverpool-Wellcome Trust Clinical Research Programme, College of Medicine, Chichiri, Blantyre, Malawi; 2 Hospital for Tropical Diseases, University College London Hospitals NHS Foundation Trust, London, UK; 3 Department of Radiology, The Royal Liverpool University Hospitals NHS Trust, Liverpool, UK; 4 Blantyre Malaria Project, Blantyre, Malawi; 5 Brain Infections Group, Walton Centre NHS Foundation Trust, and Institute of Infection and Global Health, University of Liverpool, Liverpool, UK; 6 Institute for Infection and Immunity, St George’s, University of London, London, UK; 7 Department of Clinical Sciences, Liverpool School of Tropical Medicine, Liverpool, UK; 8 Division of Infection and Immunity, University College London, London, UK

**Keywords:** HIV, Cryptococcal meningitis, Immune reconstitution inflammatory syndrome, Stroke

## Abstract

**Background:**

Cryptococcal meningitis remains the leading cause of adult meningitis in Sub-Saharan Africa. Immune Reconstitution Inflammatory Syndrome (IRIS) following anti-retroviral therapy (ART) initiation is an important complication. Here we report the first documented case of a IRIS reaction presenting as an ischemic stroke.

**Case presentation:**

A 38 year old newly diagnosed HIV-infected, ART naive Malawian male presented to a tertiary referral hospital in Blantyre, Malawi with a 2 week history of headache. A diagnosis of cryptococcal meningitis was made and the patient was started on 1200 mg fluconazole once daily and flucytosine 25 mg/kg four times daily as part of the Advancing Cryptococcal Treatment for Africa (ACTA) clinical trial. There was an initial clinical and microbiological response to anti-fungal treatment and anti-retroviral therapy was started at week 4. The patient re-presented 16 days later with recurrence of headache, fever, and a sudden onset of left sided weakness in the context of rapid immune reconstitution; peripheral CD4 count had increased from a baseline of 29 cells/μl to 198 cells/μl. Recurrence of cryptococcal meningitis was excluded through CSF examination and fungal culture. Magnetic Resonance Imaging (MRI) of the brain demonstrated multi-focal DWI (diffusion weighted imaging) positive lesions consistent with an ischemic stroke. Given the temporal relationship to ART initiation, these MRI findings in the context of sterile CSF with raised CSF protein and a rapid immune reconstitution, following an earlier favorable response to treatment is most consistent with a paradoxical Immune Reconstitution Inflammatory Syndrome.

**Conclusions:**

Stroke is an increasing cause of morbidity and mortality amongst HIV infected persons. Ischemic stroke is a recognized complication of cryptococcal meningitis in the acute phase and is thought to be mediated by an infectious vasculitis. This is the first time an ischemic stroke has been described as part of a paradoxical IRIS reaction. This report adds to the spectrum of clinical IRIS presentations recognized and highlights to clinicians the potential complications encountered at ART initiation in severely immunocompromised patients.

**Electronic supplementary material:**

The online version of this article (10.1186/s12879-018-3386-0) contains supplementary material, which is available to authorized users.

## Background

Cryptococcal meningitis (CM) remains a common opportunistic infection in settings with high HIV prevalence and is the leading cause of adult meningitis in Sub-Saharan Africa [1]. Mortality remains high in Africa, ranging from 24% [2] to 100% [3] depending upon the anti-fungal regimen used. With roll out of anti-retroviral therapy (ART), Immune Reconstitution Inflammatory Syndrome (IRIS) has emerged as a prominent early complication in CM patients. Cryptococcal IRIS (C-IRIS) is believed to be caused by the recovery of cryptococcus-specific immune responses following ART initiation resulting in a pathological inflammatory response [4]. Two distinct forms of C-IRIS are recognised: paradoxical and unmasking IRIS. Paradoxical IRIS is characterized by a clinical deterioration and severe inflammation in a patient with previously diagnosed cryptococcal disease following ART initiation despite an earlier favorable response to anti-fungal therapy [4]. Paucity of viable organisms in the CSF despite severe inflammation is characteristic of paradoxical C-IRIS [5]. In contrast, unmasking IRIS is seen in patients with sub-clinical/undiagnosed cryptococcosis presenting with meningitis soon after ART initiation [4].

C-IRIS is therefore a highly heterogeneous condition which may result in a variety of clinical syndromes with both central nervous system (CNS) and non-CNS features. CNS C-IRIS presentations reported in the literature include: aseptic meningitis and intracranial cryptococcomas [6], spinal cord abscess [7], recalcitrant raised intracranial pressure [8], optic nerve oedema [9] and cranial nerve lesions [10]. Non-CNS manifestations of C-IRIS include chorioretinitis [11], lymphadenitis [12], soft tissue abscess, hypercalcaemia, cavitatory pulmonary disease [13] and lobar pneumonitis [11].

Here we present the history of a Malawian man who developed an ischemic stroke in the context of a paradoxical C-IRIS reaction. We aim to highlight the diagnostic challenges encountered when a patient with known CM presents with recurrence of symptoms and to add to the literature describing the spectrum of clinical IRIS presentations.

## Case presentation

A 38 year old newly diagnosed HIV-infected, ART naive Malawian male presented to Queen Elizabeth Central Hospital, a large teaching hospital in Blantyre, Malawi. He reported a 2 week history of headache and vomiting. He had no past medical history of smoking, hypertension or diabetes. On examination, his blood pressure was 122/80 mmHg, pulse 124 beats/min and temperature 36.7 °C. He had a Glasgow Coma Score (GCS) of 15/15, there was evidence of meningism but no focal neurological deficit. A lumbar puncture (Additional file 1: Table S1) showed a WCC (white cell count) 6.0 × 10^6^/μL, glucose < 2.22 mmol/L (normal range 2.22–4.44 mmol/L), protein 1.68 g/L (normal range 0.15–0.45 g/L) and a positive lateral flow assay for cryptococcal antigen (crAg; IMMY, Norman, OK) (Additional file 1: Table S1). Cerebrospinal fluid (CSF) opening pressure was 28 cm H_2_0 (normal range 5–20 cm H_2_0). Quantitative cryptococcal culture (QCC) demonstrated 740,000 colony forming units (cfu) /ml (normal < 0 cfu/ml). CD4 count at admission was 29 cells/μl, Hb 11.6 g/dL, WCC 7.5 10^3^/μL, lymphocytes 0.9 10^3^/μL. A diagnosis of CM was made and the patient started on 1200 mg fluconazole once daily and flucytosine 25 mg/kg four times daily as part of the Advancing Cryptococcal Treatment for Africa (ACTA) clinical trial (Open label, multicentre randomised trial; trial no. ISRCTN45035509). The patient received 14 days of treatment as an inpatient as per protocol. He required twice daily lumbar punctures until day 7 to manage his persistently raised intra-cranial pressure (ICP). His day 7 QCC was 22,300 cfu/ml and his QCC prior to discharge on day 14 was 30 cfu/ml.

The patient re-presented 5 days post discharge with recurrence of severe headaches, neck pain, drowsiness and vomiting. On examination, he had a GCS (Glasgow coma scale) of 15/15 and had no neurological deficit. CSF opening pressure was 28 cm H_2_0 and his CSF was crAg positive, WCC 0 10^6^/μL, glucose 2.88 mmol/L and protein 1.22 g/L (Additional file 1: Table S1). The patient was commenced on amphotericin to treat suspected CM relapse whilst awaiting cryptococcal culture. The culture was subsequently negative and amphotericin was discontinued. He continued on 800 mg fluconazole once daily and was discharged 19 days later with no ongoing symptoms of meningitis. ART was started according to national guidelines (TDF/3TC/EFV) at day 39.

The patient re-presented to hospital at day 54 (16 days post ART commencement) with recurrence of headache and fever, and a sudden onset of left sided weakness. He reported adherence to his fluconazole and ART. On examination, temperature was 38.2 °C, blood pressure 135/92 mmHg, pulse 89 beats/min, respiratory rate 22 breaths/min. He had a new left sided hemiparesis involving both the upper limb and lower limb (power 4/5 using the Medical Research Council (MRC) scale), and there was no sensory deficit. The CSF opening pressure was 21 cm H20. The CSF crAg remained positive, CSF WCC 0 10^6^/μL, CSF protein was high, 3.26 g/L. Culture remained negative (Additional file 1: Table S1).

**Fig. 1:**
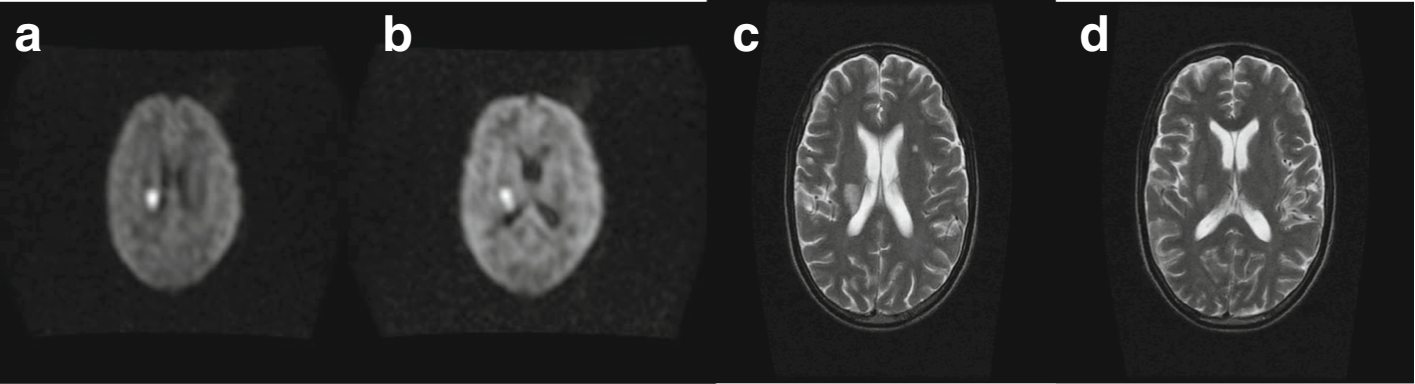
**a**-**d** are MRI sequences taken on day 5 of an acute presentation of left arm and leg weakness; **a**-**b** are DWI axial B-900 sequences, showing restriction in the right corona radiata (**a**) and posterior limb of the internal capsule (**b**). **c** and **d** (T2 axial sequences) showed high signal in the corresponding areas to the DWI lesions; consistent with an acute ischemic stroke. High T2 signal was also seen in the left deep white matter (**c**); Not shown but there was increased T2 signal (with DWI restricted diffusion) in the right cerebral peduncle and the brainstem (without DWI changes); suggesting further ischaemia

His peripheral CD4 count had increased from a baseline of 29 cells/μl to 198 cells/μl. Magnetic Resonance Imaging (MRI) of the brain (performed on day 5 of symptom onset) demonstrated multi-focal DWI positive lesions in the right corona radiata (Fig. 1), right posterior limb of the internal capsule, right cerebral peduncle and 1 month later, the right caudate nucleus; these changes were consistent with an acute ischemic stroke. There was high T2 signal without DWI restricted diffusion in the right brainstem and left deep white matter, suggesting previous ischemia. The MRI findings and raised CSF protein in the context of rapid immune reconstitution, following an earlier favorable clinical and microbiological response to anti-fungal treatment and a sterile CSF is consistent with paradoxical C-IRIS as opposed to acute CM infection. Because of the involvement of both anterior and posterior circulation, this met the criteria for probable cerebral vasculitis^12^. The 0.35 T GE MRI scanner used, does not support an ADC sequence. However, given the sudden onset of the symptoms, these focal DWI changes, in the corresponding anatomical location, occurring within a vascular territory, the etiology is unlikely to be anything other than vascular.

The development of an ischemic stroke was considered to be either a late manifestation of the CM infection or due to intensification of HIV- or CM- associated inflammation with immune reconstitution^8^. Fluconazole at a dose of 400 mg was continued with ART and the patient achieved good functional improvement. Corticosteroids were not given.

**Fig. 2:**
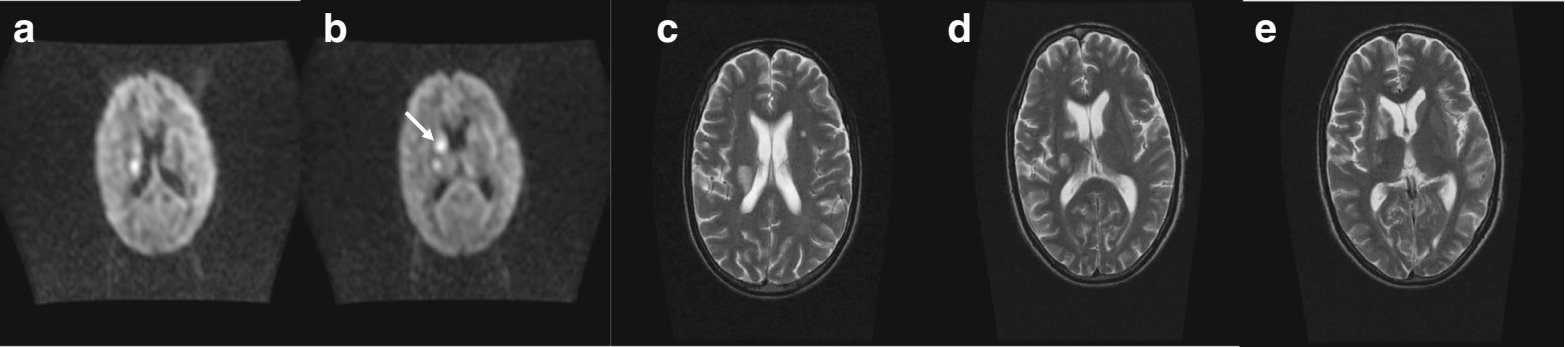
**a**-**e** are MRI sequences taken 1 month later from symptom onset and on the background of a resolved left sided weakness. **a**-**b**; shows a new DWI lesion (**b** – arrow) suggesting evolving inflammation. However, the burden of the previous DWI changes reported in (1) is less. **c**-**d** (T2 axial) shows maturation of previously reported infarct

An MRI scan performed one month later demonstrated maturation of the ischemic stroke (Fig. 2), and a new DWI lesion in the right caudate, consistent with C-IRIS. The patient however reported complete resolution of the left sided weakness and remained well at day 185.

## Discussion and conclusions

This case demonstrates the diagnostic challenges encountered when a patient with known CM presents with recurrence of symptoms following commencement of ART. Paradoxical C-IRIS complicates around 15% of cases of CM in ART naïve patients [14], usually during the first ten months of ART [4]. There is no definitive diagnostic test for IRIS but by adopting the clinical criteria checklist approach set out by the International Network for the Study of HIV-associated IRIS [4] (Additional file 1: Table S2), we were able to diagnose paradoxical C-IRIS in this low resource setting. This patient had several of the recognized risk factors for paradoxical IRIS including: low CD4 count at ART initiation, high fungal burden [15] and lack of initial CSF inflammation (WBC < 25 cells/μl and CSF protein < 50 mg/dL) [16]. These characteristics are not uncommon in patients with CM in Sub-Saharan Africa.

In line with current treatment guidelines, ART for this patient was not commenced until after week 4 of treatment, and was continued despite the diagnosis of paradoxical C-IRIS [17]. Although the reported mortality risk associated with C-IRIS is highly variable (0–83% [4]), ART and successful immune reconstitution is key to survival in HIV and so ART was not interrupted or discontinued due to IRIS. For non-life threatening IRIS manifestations, there are no specific definitive treatments recommended and symptoms should resolve spontaneously within weeks to months. For deteriorating patients with CNS inflammation leading to raised intra-cranial pressure, with no alternative diagnosis, corticosteroids should be considered (0.5 mg – 1.0 mg/kg per day of prednisolone) [17]. As our patient was clinically stable with improving neurology, steroids were not given.

Ischemic stroke is a recognized complication of CM in the acute phase and is thought to be mediated by an infectious vasculitis, primarily causing an endarteritis obliterans [18]. In this case however, the progressive parenchymal disease in the presence of sterile CSF and absence of traditional vascular risk factors, is most consistent with an immuno-pathological process. The increased risk of cerebrovascular disease in patients with HIV is well documented [19]. The pathophysiology is likely multifactorial with proposed mechanisms including increased prevalence of traditional vascular risk factors, infectious vasculitis, HIV-associated vasculopathy, and coagulopathy [20]. In a case-control study of 222 Malawian adults with MRI confirmed acute stroke, the highest risk of stroke in patients with untreated HIV infection was in the first 6 months after starting antiretroviral therapy (ART) (aOR 15.6 [4.21–46.6], *p* < 0.001) [19]. These results have led to the hypothesis that IRIS may be contributing to the disease.

In conclusion, this case report adds to the spectrum of clinical IRIS presentations encountered while managing CM. Whilst ART is essential in improving survival outcomes in these patients, clinicians should be aware of the complications of ART initiation including C-IRIS and stroke.

## Additional files

Additional file 1: Supplemental tables. (DOCX 17 kb)

## Abbreviations

ART: Anti-retroviral therapy
C-IRIS: Cryptococcal – immune reconstitution inflammatory syndrome
CM: Cryptococcal meningitis
CNS: Central nervous system
CSF: Cerebrospinal fluid
DWI: Diffusion weighted imaging
GCS: Glasgow coma scale
HIV: Human immunodeficiency virus
ICP: Intra-cranial pressure
MRC: Medical research council
MRI: Magnetic Resonance Imaging
QCC: Quantitative cryptococcal culture
