# Supplementary material for: Ischemic stroke as a complication of cryptococcal meningitis and immune reconstitution inflammatory syndrome: a case report
Source: BMC Infect Dis. 2018 Oct 16;18:520. doi: 10.1186/s12879-018-3386-0 (PMC6192067; doi:10.1186/s12879-018-3386-0)
Supplement: Supplementary file 1 — Supplemental tables. (DOCX 17 kb) [file 12879_2018_3386_MOESM1_ESM.docx]

Additional file 1: **Table S1**. Serial quantitative cryptococcal cultures (QCC), cryptococcal antigen test (crAg), CSF white blood cell count, CSF glucose, CSF protein and peripheral CD4 cell count.

|  | **QCC (cfu/ml)** | **crAg** | **CSF WBC (10^6/uL)** | **CSF Glucose**  **(mmol/L)** | **CSF Protein**  **(g/L)** | **Peripheral CD4 count**  **(cells/µl)** |
| --- | --- | --- | --- | --- | --- | --- |
| **Day 1** | 740,000 | + | 0 | 3.67 | 3.26 | 29 |
| **Day 7** | 70,500 |  |  |  |  |  |
| **Day 14** | 30 |  |  |  |  |  |
| **Readmission at day 19** | 0 | + | 0 | 2.88 | 1.22 |  |
| **Readmission at day 54** | 0 | + | 0 | 3.67 | 3.26 | 198 |

Additional file 1: **Table S2**. Case definition for paradoxical cryptococcal immune reconstitution inflammatory syndrome

| Antecedent requirements:Taking ARTCryptococcal disease diagnosed before ART by positive culture or typical clinical features with positive India ink staining or antigen detection.Initial clinical response to antifungal therapy with partial or complete resolution of symptoms or signs, fever or other lesions, or reduction in CSF cryptococcal antigen concentration or quantitative culture. |
| --- |
| Clinical criteria:Event occurs within 12 months of ART initiation, reintroduction, or regimen switching after previous failure.Clinical disease worsens with one of the following inflammatory manifestations of cryptococcosis:MeningitisLymphadenopathyIntracranial space occupying lesion or lesionsMultifocal diseaseCutaneous of soft tissue lesionsPneumonitis or pulmonary nodules |
| Other explanations for clinical deterioration to be excluded:Non adherence or suboptimum antifungal therapyAlternative infection or malignant disease in the affected siteFailure to ART excluded if possible |
